# Surgical Site Infections Complicating the Use of Negative Pressure Wound Therapy in Renal Transplant Recipients

**DOI:** 10.1155/2019/2452857

**Published:** 2019-09-24

**Authors:** Susanna Lam, Ngee-Soon Lau, Jerome Martin Laurence, Deborah Jean Verran

**Affiliations:** ^1^Royal Prince Alfred Hospital, Department of Transplantation Services, Sydney, Australia; ^2^The University of Sydney, Faculty of Medicine and Health, Edward Ford Building, Fisher Road, NSW 2006, Australia

## Abstract

Surgical site infections (SSI) of the abdominal wall in renal transplant recipients can on occasion require management with negative pressure wound therapy (NPWT). This is often successful, with a low risk of further complications. However, we describe three cases in which persistent or recurrent surgical site sepsis occurred, whilst NPWT was being deployed in adults with either wound dehiscence or initial SSI. This type of complication in the setting of NPWT has not been previously described in renal transplant recipients. Our case series demonstrates that in immunosuppressed transplant recipients, there may be ineffective microbial or bacterial bioburden clearance associated with the NPWT, which can lead to further infections. Hence recognition for infections in renal transplant patients undergoing treatment with NPWT is vital; furthermore, aggressive management of sepsis control with early debridement, antimicrobial use, and reassessment of the use of wound dressing is necessary to reduce the morbidity associated with surgical site infections and NPWT.

## 1. Introduction

In adult renal transplant recipients, the rate of reported surgical site infections (SSI) varies from 4.8–18.6% [1–5], which can add considerable morbidity to the management of these patients [6]. SSI can also supervene particularly when the integrity of the abdominal wall is lost in the setting of superficial and, or deep fascial dehiscence [7]. Furthermore, the requirement for immunosuppression along with a number of underlying medical comorbidities in renal transplant recipients may contribute to the incidence of SSI in this population [5, 8, 9].

Negative pressure wound therapy (NPWT), or vacuum assisted closure (VAC) therapy, has become a useful adjunct in the management of wound complications or SSI in orthopedic trauma and general surgery [10–14], including for SSI involving prosthetic mesh [15, 16]. Favorable outcomes with the use of NPWT for abdominal wall complications and SSI have been reported in renal transplant recipients to date [17, 18]. However, no data have been reported for persistent or recurrent surgical site infection supervening as a result of the use of the NPWT for wound complications in adult renal transplant recipients. Hence, we report in detail on three renal transplant recipients who developed recurrent or persistent wound infections, whilst being managed for abdominal wall surgical site complications with NPWT.

At our institution, the standard approach to managing abdominal wall complications after renal transplant detected clinically or radiologically is to drain, debride, and clean the wound at the bedside or in the operating theatre and then apply the NPWT.

All wounds which were suspected to be clinically infected are all initially swabbed and the swabs sent for microscopy, culture, and sensitivities (MCS) analysis. Antimicrobial therapy was given empirically in the setting of acute onset of SSI with evidence of spreading infection, and then tailored according to culture sensitivities or local sensitivity patterns, and was decided on a case by case basis and with infectious disease service input.

Application of the NPWT at our institution involves using black polyurethane foam, which is cut to size and applied to the wound and then sealed using an occlusive dressing at a continuous negative pressure of 125 mmHg connected to an “ActiVac” Therapy system (Vacuum Assisted Closure, VAC Therapy KCI Medical Pty Ltd USA [19]). Dressings and foam are then changed twice weekly as an inpatient or if appropriate in the transplant outpatient clinic. Wounds are reviewed at the time of dressing change by the treating surgical team to assess progress, and the NPWT is discontinued once wounds are shallow enough to be suitable for simple daily dressing changes.

## 2. Case Presentations

### 2.1. Case 1

A 68-year-old female with a BMI of 34 kg/m^2^ and a history of polycystic kidney disease, underwent a deceased donor renal transplant in December 2017, with initial graft function being obtained. Three days post-transplant, the recipient developed a serous discharge at the supero-lateral aspect of the wound, secondary to the presence of a subcutaneous abdominal wall collection. The supero-lateral aspect of the hockey stick surgical wound was opened, a microbiology swab taken and sent for MCS, followed by wound irrigation with a copious amount of normal saline along with wound debridement at the bedside until the wound itself was clean, with a healthy intact base. A tapered piece of black foam was placed in the lateral aspect of the surgical wound, and the NPWT was applied at a continuous negative pressure of 125 mmHg, noting that the skin over the infero-medial aspect of the wound remained intact. The microbiology swab grew coagulase negative *Staphylococcus*, *Corynebacterium* species, *Enterococcus* species, and *Prevotella* species. The recipient was treated with intravenous cefazolin for four days and then oral cephalexin for a week. Repeat wound culture swabs were negative after 1 further week, at which stage the open wound appeared healthy to clinical examination.

The NPWT was continued in the community due to slow wound healing with twice-weekly dressing changes, with the wound initially appearing clean, with a healthy intact wound base, with no other clinical signs of infection during regular surgical team review. However, on day 24 post-transplant, the recipient developed evidence of an additional SSI, with new tenderness to clinical palpation at the inferno-medial aspect of the wound. However, the supero-lateral aspect of the wound base on clinical examination, after removal of the foam, appeared clean. Hence a computed tomography (CT) scan of the abdomen and pelvis was performed, which showed a small subcutaneous collection in the medial aspect of the abdominal wall, but separate to the NPWT foam. This collection was then aspirated percutaneously under ultrasound guidance. The culture of the aspirated fluid revealed a scanty growth of *Enterococcus* species and *Prevotella bivia*. The recipient was treated with intravenous cefazolin for 3 days, followed by oral amoxicillin and clavulanic acid for a further 5 days. The recipient had sustained normal graft function, and NPWT was overall tolerated well. Complete abdominal wall healing was confirmed at 82 days post-transplant.

### 2.2. Case 2

A 59-year female with a BMI of 30.1 kg/m^2^ and a background of diabetes and hypertension underwent a deceased donor renal transplant in November 2017. She then had delayed graft function, requiring haemodialysis within the first week. At 19 days post-transplant, the recipient developed a new clinically evident superficial abdominal wall collection above the deep fascia. There were no signs of spreading infection. The wound was re-opened at the bedside, swabbed for MCS, washed out with copious saline irrigation, and debrided until clean, and the wound base was clean and intact with evidence of granulation. The NPWT with black foam was applied at −125 mmHg continuous pressure. This initial wound swab MCS was negative for organisms at this stage. The dressings were changed twice a week, first as an inpatient, and then as an outpatient, the wound appeared to be clean with a healthy base on dressing changes.

However, on day 59 post-transplant, during a routine dressing change, a new collection of turbid fluid was detected beneath the foam, anterior to the deep fascia. This wound appeared infected, with slough at the base; therefore, it required copious washout and debridement in the operating theatre, until the wound base appeared clean and intact and the NPWT was then reapplied. This wound swab MCS revealed a penicillin sensitive *Staphylococcus aureus*, and the recipient was treated with oral flucloxacillin.

At 79 days post-transplant, the recipient developed urinary sepsis and acute kidney injury along with a concurrent abscess in the abdominal wall, deep to the NPWT foam, which was detected on CT scan of the abdomen and pelvis. The NPWT dressings were immediately removed at the bedside, the wound appeared infected, with abscess and was again swabbed and washed out. The NPWT was abandoned due to recurrent abscess formation around the foam. This was then replaced with a regimen of simple gauze dressings. The wound swab MCS grew *Staphylococcus aureus*, mixed skin flora and coliforms, whilst the urine MCS was positive for *Escherichia Coli* and *Klebsiella pneumoniae*. The recipient received empirical intravenous tazobactam and piperacillin (Tazocin) and changed to ceftriaxone based on sensitivities, for a total of 2 weeks. The wound subsequently healed at 109 days post-transplant with a combination of gauze dressings and antibiotics. By this stage the recipient had ongoing normal allograft function.

### 2.3. Case 3

A 55-year female with a BMI of 42.3 kg/m^2^ and a background of IgA nephropathy underwent a deceased donor renal transplant in January 2017. She had a spontaneous fall in serum creatinine within 72 h, and was initially making an uncomplicated recovery. However, when lower abdominal symptoms developed, a deep fascial dehiscence along with a superficial fluid collection was detected clinically and then confirmed with CT imaging of the abdomen and pelvis. This required surgical debridement and operative repair on post-transplant day 20. The abdominal fluid collection appeared turbid and was swabbed for MCS, furthermore deep fascial closure was not possible due to a combination of tissue oedema and tissue loss. Therefore, a tension free repair with a dual layer vicryl and prolene onlay mesh was performed and the skin was closed primarily with interrupted nylon sutures. The abdominal wall culture was positive for *Candida albicans*, and the recipient was scheduled to be treated with oral fluconazole for three months.

On post-transplant day 59, the recipient developed a new serous discharge from the skin at the surgical site, secondary to a clinically detected superficial collection. This required reoperative surgery with a thorough wash out; unhealthy tissue within the wound was debrided until the tissue at the wound base was clean and intact. The NPWT dressing was applied, with black foam at continuous pressure of −125 mmHg, to the surgical site. A repeat wound swab performed intraoperatively revealed scanty growth of *Candida albicans*, requiring the ongoing prescription of fluconazole. The tissue specimen taken during the debridement returned a negative MCS. The NPWT dressing was changed twice a week, and the wound appeared to be healing well, such that to facilitate delayed primary closure of the healing superficial aspect of the surgical site, it was progressively closed with delayed skin sutures and downsizing, and removal of the foam by post-operative day 105.

Five days following the NPWT removal, the recipient re-presented with new clinical signs of sepsis, and an urgent CT scan confirmed an abscess within the subcutaneous tissue of the surgical site. This required operative management, and the superficial wound was reopened, swabbed, and unhealthy, devitalized tissue was debrided along with a copious wash out being performed. Of note, the prosthetic mesh had healed into the deep abdominal wall; hence, the foam and NPWT were reapplied superficial to the mesh. This was tolerated well. The recipient received intravenous Tazocin, and was stepped down to oral amoxicillin and clavulanic acid combined with metronidazole and fluconazole for 3 weeks. The wound MCS on delayed tissue culture on enrichment growth revealed a *Corynebacterium* species 15 days following this repeat surgery. Histopathology of the tissue also revealed fat necrosis and a foreign body reaction. Hence no attempt was made to close the skin with sutures again. The recipient had sustained normal graft function, and the surgical site was sufficiently healed with the use of NPWT by 201 days post-transplant.

## 3. Discussion

We report three cases of renal transplant recipients who despite being managed in a standard manner with a NPWT dressing regimen for abdominal wall complications, have then all gone on to either develop recurrent infection and or further episodes of sepsis in the abdominal wall, with at times different organisms being involved. This particular complication has not been previously described in the literature, in the context of NPWT being used to manage a spectrum of abdominal wall surgical site complications in renal transplant recipients [17, 20–22] and including previously in our own unit [23].

Up to now NPWT has been used successfully to manage contaminated or infected wounds with generally minimal complications [10, 24, 25]. There are infrequent reports of NPWT complications in the form of recurrent, clinically significant wound infections in other settings; hence, this entity is most likely underreported in the literature. Although there are reports of the bacterial bioburden not being reduced despite the use of NWPT, in most cases the wounds continue to heal without incident [14]. There are a limited number of reported cases of infectious complications associated with the use of NPWT including toxic shock syndrome [26], sepsis associated with burn wounds following eschar debridement [27], delayed sepsis in a blast injury wound [28], along with abscess, and sepsis in acute and chronic wounds treated with NPWT [29]. Moreover, the Food and Drug Administration in the United States have reported a range of complications associated with NPWT including predominantly haemorrhage and complications related to retained foam in patients being treated with NPWT [30]. Infection is mentioned as a complication in 27 cases but there are no other data on the context or nature of the recurrent infections.

Although the mechanisms associated with the onset of infectious complications with the use of NPWT in our cases are not totally clear, the immunosuppressed state of renal transplant recipients may predispose them to developing further sepsis, particularly in the setting of persistent microbial colonisation of the wound, which can occur despite the use of NWPT [14, 31, 32]. Whilst NPWT has been shown to improve wound healing by reducing the bacterial load in animal models [33], this is not a consistent finding [31, 32, 34]. In vitro models of tissue treated with NPWT or foam from the NPWT did not show a decrease in bacterial load [35, 36], whilst other clinical studies have shown an increase in the quantitative counts of bacteria [14], and has also been confirmed clinically in a series of patients being managed with NWPT following a laparotomy for sepsis [37]. Furthermore, alterations in the bacterial flora of the wound can lead to the proliferation of some sub types, such as *staphylococcus aureus* [10, 11, 26, 32], which was evident in Case 2.

Our three cases who were all transplanted with allografts from deceased donors, sustained further infectious complications associated with a range of organisms including *Staphylococcus aureus*, *Corynebacterium* species, and fungi. These are typical of organisms commonly isolated from SSI, which occur within the first postoperative month time frame in renal allograft recipients [38]. Moreover, changes in pathogens and an increase in different bacterial isolates have been found in wounds treated with NPWT [39]. There is also evidence that some gram positive organisms may persist in an open wound despite the use of NWPT, whilst the numbers of non-fermentative types of gram negative bacilli are reduced [32]. Of note, Case 3 had an unusual surgical site infection associated with candida, and although a donor derived infection is a possibility [40], the source of this infection was never formally determined. Although the presence of mesh could have potentially led to additional problems with infection, there is now evidence for the use of prosthetic mesh as part of the management of complicated wounds in the abdominal wall. This has been reported in a cohort of patients following laparotomy, deployment of NWPT can facilitate abdominal wall healing over a contaminated mesh repair [16]. Case 2 was the only case where urinary sepsis occurred at the same time as the SSI associated with the NPWT foam. Whilst there is no apparent correlation between the urine and SSI infections, it is possible that the net immunosuppressed state of the recipient had increased their vulnerability to pathogens and infections [38].

One of the possible risks for the development of SSI with NPWT is that the polyurethane foam acts as a foreign body, generating an inflammatory reaction in the wound [41], as well as impacting on wound healing by failing to reduce the extent of bacterial colonization [42]. There have been recent reports of the bacterial burden not being altered by the use of NWPT [31]. This bacterial burden is only reduced in patients in whom aggressive wound management involving surgical debridement, irrigation, and judicious use of antibiotics is undertaken as was required in our 3 cases [43]. The foreign body reaction also observed in the third case is suggestive of an altered mechanism of wound healing as an underlying facilitator of ongoing SSI, despite abdominal wall healing initially being obtained with NPWT on the first occasion.

Obesity is a known risk factor for abdominal wall complications in renal transplant recipients [4], and it is possible that it may also be associated with further SSI in the setting of NPWT noting the average BMI of 35.6 kg/m^2^ in our cases. It is also postulated that in obese patients, the abdominal pannus may have a traction effect which alters the forces across the surgical wound, between the supine and erect positions, which are not mitigated by the NPWT alone.

This case report is beneficial in highlighting the unusual complication of further SSI that may be associated with the use of NPWT dressings in renal transplant recipients and the prolonged time then taken to achieve full healing of the abdominal wall. This can add to the significant challenges associated with managing ongoing abdominal wall complications in the context of obesity, immunosuppression, and prolonged wound healing. The potential for this additional complication also necessitates that clinical vigilance be maintained for adult recipients in whom NPWT is being utilized. Furthermore, we highlight the need to tailor the management approach to each patient, by using a combination of bedside management, radiological, and surgical approaches, as required.

We also highlight the added financial cost and burden of managing ongoing surgical site infection with the ensuing requirement for prolonged wound management for periods of up to 180 days requiring further procedures in hospital, along with further use of the NPWT and or, other dressings. Use of the NPWT device with two foam dressing changes costs up to $AUD 500 per week, or approximately $AUD 7,000 for the average NPWT usage of 102 days for the device consumables, in addition to the cost of staff time spent on managing the dressings and performing procedures, the cost of additional antibiotics plus the extra in-hospital stay costs. However, being able to deliver NPWT as an outpatient can save on additional inpatient costs. This was an approach utilised in all three of our cases, as previously described in a group of patients with complex abdominal wall surgical site issues following general surgical procedures [44].

This case report is limited by the small sample size and its retrospective nature. There is also potential for false positives in wound culture, although wounds were reviewed regularly for signs of infection and managed based on clinical findings. The true effect of NPWT and the associated risk of complications related to its use in renal transplant recipients will require further collaborative prospective studies. It is apparent that the use of NPWT may not clear a wound of bacterial contamination, and there is an ongoing risk of further bacterial proliferation and possible infection in the context of NPWT [32]. Moreover, there is a complex interplay between patient factors, the net state of immunosuppression and bacterial bioburden and virulence in successful wound healing [38]. Therefore, identifying potential risk factors for SSI associated with the use of NPWT is required. The prophylactic use of combined negative pressure wound plus instillation therapy with antiseptics, or the use of silver impregnated foam are potential strategies that may now also be used to treat contaminated wounds in renal transplant recipients [45–47].

## 4. Conclusion

The management of surgical site infections and wound breakdown in adult renal transplant recipients is challenging. The use of NPWT can often successfully promote wound healing of the open abdominal wall in this particular cohort. However, our case series demonstrates that in immunosuppressed transplant recipients, there may be ineffective microbial or bacterial bioburden clearance associated with the NPWT and antimicrobial use, which can lead to further infections ensuing. As such, when an infection is detected, we recommend sepsis control with antimicrobial therapy in conjunction with early and aggressive surgical debridement for control of sepsis and reassessment of the dressing strategy, which may or may not involve further use of NPWT.

## Abbreviations

AUD: Australian dollars
BMI: Body mass index
CT: Computed tomography
MCS: Microscopy culture sensitivities
NPWT: Negative pressure wound therapy
SSI: Surgical site infection
VAC: Vacuum assisted closure.
